# Hepatitis C Virus Core Antigen Test in Monitoring of Dialysis Patients

**DOI:** 10.1155/2012/832021

**Published:** 2012-12-04

**Authors:** Gioacchino Li Cavoli, Carmela Zagarrigo, Onofrio Schillaci, Francesca Servillo, Angelo Tralongo, Mario Coglitore, Filippo Spadaro, Concetta Scimeca, Natalia Li Destri, Ugo Rotolo

**Affiliations:** ^1^Nephrology and Dialysis, ARNAS Civico and Di Cristina Hospital, Via Francesco Cilea 43, 90144 Palermo, Italy; ^2^Microbiology and Virology, ARNAS Civico and Di Cristina Hospital, Palermo, Italy

## Abstract

Hepatitis C virus infection is a persistent worldwide public health concern. The prevalence of HCV infection is much higher in patients on chronic haemodialysis (HD) than in the general population. HCV infection can detrimentally affect patients throughout the spectrum of chronic kidney disease. Despite the control of blood products, hepatitis C virus transmission is still being observed among patients undergoing dialysis. Detection systems for serum HCV antibodies are insensitive in the acute phase because of the long serological window. Direct detection of HCV depends on PCR test but this test is not suitable for routine screening. Recent studies have highlighted the importance of HCV core antigen detection as an alternative to PCR. Few studies exist about the efficacy of HCV core antigen test in dialysis population. We studied the utility of HCV core antigen test in routine monitoring of virological status of dialysis patients. We screened 92 patients on long-term dialysis both by PCR HCV-RNA and HCV core antigen test. The sensitivity of HCVcAg test was 90%, the specificity 100%, the positive predictive power 100%, the negative predictive power 97%, and the accuracy 97%. We think serological detection of HCV core antigen may be an alternative to NAT techniques for routine monitoring of patients on chronic dialysis.

## 1. Introduction

Hepatitis C virus (HCV) infection is a persistent public health concern. HCV infects approximately 170 million people worldwide [1]. The prevalence of HCV infection is much higher in patients on chronic haemodialysis (HD) than in the general population. The estimated prevalence of HCV infection in HD patients is 7.8% in the USA [2], 5.2% in Germany [3], and 10% in Japan [4]. A recent study from Australia and New Zealand [5] in HD patients (*n* = 23,046) reported an independent and significant association between anti-HCV positive serologic status and all-cause mortality over a 10-year followup (HR, 1.25, 95% CI 1.07–1.46, *P* = 0.004). Despite the control of blood products, HCV transmission is still being observed among HD patients. HCV infection is usually diagnosed based on the detection of anti-HCV antibody, while it goes undetected in the first 4–6 weeks of infection (so-called window period). Furthermore, patients positive for anti-HCV antibody include both those who are actively infected and those who have recovered from infection [6]. Kidney Disease Improving Global Outcomes (KDIGO) clinical practice guidelines for the prevention, diagnosis, evaluation, and treatment of hepatitis C in chronic kidney disease [7] recommended the use of nucleic acid amplification technology (NAT). A quantitative HCV core antigen (HCVcAg) test has been developed for the confirmation of viremia in patients with hepatitis C. This test can detect total nucleocapsid core antigen whose sequence is highly conserved across HCV genotypes. Some studies in the general population have highlighted the importance of HCVcAg detection as an alternative to NAT for early diagnosis of infection, as direct marker of viral replication in chronic phase of infection and as relevant marker for predicting and monitoring the response to therapy [8]. Few studies exist about the efficacy of HCVcAg test in patients on chronic HD in the early diagnosis of HCV infection [9–11]. The aim of this study is to apply these diagnostic advances in routine monitoring of chronic dialysis patients.

## 2. Materials and Methods

From September 2009 to February 2010 in our dialysis ward we included NAT and HCVcAg testing in the current clinical practice. Then we reviewed the medical records evaluating the virological monitoring. We evaluated 92 patients on long-term dialysis; among these subjects, 67 were on haemodialysis three times per week and 25 on peritoneal dialysis. We evaluated HCVcAg and HCVAb by chemiluminescent assay (Architect Abbott), HCV immunoblotting by InnoLia, HCVRNA by PCR (TaqMan Roche), viral genotype by INNO-LiPA 2.0, and other routine tests. Demographic and clinical characteristics of screened patients are given in Table 1.

## 3. Results

We detected 66 HCVAb-negative and 26 HCVAb-positive patients. All HCVAb-negative subjects were both HCV-RNA negative and HCVcAg negative. Among 26 HCVAb-positive patients, 6 were both HCVcAg negative and HCV-RNA negative: 4 of these were immunoblotting negative and 2 immunoblotting positive; we considered in these 6 patients the current absence of HCV infection as in HCVAb negative subjects. Finally serum HCVRNA was detectable in 20 HCVAb-positive patients (Table 2). The results of liver function tests were unremarkable.

Among 20 HCVRNA positive subjects, 2 were HCVcAg negative and 18 positive; the minimum HCVRNA IU/HCVAg pg ratio was 78; the maximum was 425.000 and the mean was 64.401. Therefore, in 18 out of 92 screened patients, HCVcAg test was positive (Table 3).

## 4. Discussion

HCV infection continues to be the most frequently recognized cause of liver damage in patients with CKD [12]. Although a severe clinical course of HCV-related liver disease seems unusual in most HD patients and cirrhosis is an infrequent event among dialysis patients, longitudinal studies have found an independent and significant relationship between anti-HCV antibody positivity and reduced patient survival [13, 14].

The Dialysis Outcomes and Practice Patterns Study (DOPPS) on HD patients in three continents [15] had reported an independent and significant association between positive anti-HCV antibody and mortality risk (adjusted relative risk, 1.17; *P* < 0.0159).

Fabrizi et al. showed that HCV-seropositive HD patients had higher rates of liver disease-related death than their seronegative counterparts, but that cardiovascular and infectious disease-related mortality rates were similar [16]. Ohsawa et al. showed that seropositivity for anti-HCVcAg is independently associated with increased all-cause, cardiovascular, and liver disease-related mortality in HD patients [17].

It is important to diagnose a hepatitis C virus infection in the acute phase in order to reduce the incidence of this infection in high-risk populations like HD patients [18]. Biochemical evaluation of HCV infection in patients with CKD is inaccurate. Serum aminotransferase values are typically lower in dialysis patients than the nonuremic populations [19]. Detection systems for serum HCV antibodies are insensitive in the acute phase because of the long serological window. The direct detection of HCV depends on NAT techniques with several problems: frequent unavailability, considerable skill requirement, limited reproducibility, and overall important costs. HCV detection by PCR-RNA, although widely accepted as a gold standard test in the diagnosis of HCV infection in CKD patients, is not suitable for routine screening. Recently HCVcAg quantification assay has proved useful for an early diagnosis of HCV infection in community-based and in dialysis populations. HCVcAg may be an alternative to HCV-RNA detection, since no subjects, who were negative for HCVcAg, were positive for HCV-RNA in a large population-based cohort study of Ohsawa et al. [20]; a recent study of Kato et al. also suggests that detection of HCVcAg combined with anti-HCV antibody is useful in predicting long-term survival prognosis of persistent HCV infection in HD patients [21].

According to our experience HCVcAg test is both a cost-effective (a single sample has a 120$ charge for PCR HCVRNA and a 20$ charge for HCVAg test) and a less labour-intensive alternative to NAT tests. These features make this assay useful for routine of chronic dialysis treatment patients.

## 5. Conclusions

Serological detection of HCVcAg may be an alternative to NAT techniques for routine monitoring of patients on chronic dialysis towards the prevention of HCV spread. It is an accurate marker for early identification of HCV infection; it can improve virological monitoring and integrate the diagnosis of acute hepatitis C in dialysis population. The minimal cost and its easiness make this assay useful for routine long-term dialysis treatment patients. These findings suggest that HCVcAg is applicable for clinical use as an alternative to NAT test.

## Figures and Tables

**Table 1:** Demographic and clinical characteristics of 92 patients screened.

Males/females	54/38
Age (mean)	68.6 years
Haemodialysis/peritoneal dialysis	67/25
Caucasian	88
Asiatic/African	2/2
HBsAg-positive/HIV-positive	2/1
Primitive renal disease	
Hypertensive nephropathy	31
Diabetes mellitus	26
Polycystic kidneys	9
Chronic glomerulonephritis	12
Tubulointerstitial diseases	6
Other	4
Undetermined	2

**Table 2:** Principal features of 20 HCVRNA positive patients.

Genotype	
1b	19
2	1
HCVcAg positive	18
HBsAg-positive	2
HBsAb-positive or HBcAb-positive	18
HIV-Ab positive	1
Haemodialysis/peritoneal dialysis	17/9

**Table 3:** Comparison between HCVcAg and PCR HCV-RNA test.

HCVcAg test	PCR HCV-RNA test		Total
Result	Positive	Negative	
Positive	18	0	18
Negative	2	72	74

Total	20	72	92

The sensitivity of HCVcAg test was 90%, the specificity 100%, the positive predictive power 100%, the negative predictive power 97%, and the accuracy 97%.
